# Socioeconomic characterization of regions through the lens of individual financial transactions

**DOI:** 10.1371/journal.pone.0187031

**Published:** 2017-11-30

**Authors:** Behrooz Hashemian, Emanuele Massaro, Iva Bojic, Juan Murillo Arias, Stanislav Sobolevsky, Carlo Ratti

**Affiliations:** 1 Senseable City Lab, Massachusetts Institute of Technology, Cambridge, MA, United States of America; 2 HERUS Lab, Institute of Environmental Engineering (ENAC), École Polytechinque Fédérale de Lausanne (EPFL), CH-1015 Lausanne, Switzerland; 3 Singapore-MIT Alliance for Research and Technology, Singapore, Singapore; 4 Data & Analytics, BBVA, Madrid, Spain; 5 Center For Urban Science and Progress, New York University, Brooklyn, NY, United States of America; 6 Institute Of Design And Urban Studies of The Saint-Petersburg National Research University Of Information Technologies, Mechanics And Optics, Saint-Petersburg, Russia; University of Oxford, UNITED KINGDOM

## Abstract

People are increasingly leaving digital traces of their daily activities through interacting with their digital environment. Among these traces, financial transactions are of paramount interest since they provide a panoramic view of human life through the lens of purchases, from food and clothes to sport and travel. Although many analyses have been done to study the individual preferences based on credit card transaction, characterizing human behavior at larger scales remains largely unexplored. This is mainly due to the lack of models that can relate individual transactions to macro-socioeconomic indicators. Building these models, not only can we obtain a nearly real-time information about socioeconomic characteristics of regions, usually available yearly or quarterly through official statistics, but also it can reveal hidden social and economic structures that cannot be captured by official indicators. In this paper, we aim to elucidate how macro-socioeconomic patterns could be understood based on individual financial decisions. To this end, we reveal the underlying interconnection of the network of spending leveraging anonymized individual credit/debit card transactions data, craft micro-socioeconomic indices that consists of various social and economic aspects of human life, and propose a machine learning framework to predict macro-socioeconomic indicators.

## Introduction

Since the advent of pervasive digital technology, people are ubiquitously interacting with their environment and leaving digital traces of their daily activities, from when they are commuting to work by taking a bus or driving their own car to when they are socializing by making a call or posting to social media. Many of these traces contain geolocated information, which enables identifying location and time of a certain activity, recorded through various technologies such as mobile phones, wearables, connected cars, social media, and credit cards. This geolocated data has created a great potential for studies on human mobility [1, 2], social phenomena [3–6], epidemic outbreaks [7], healthcare [8], urban structure [9], etc.

Among these datasets, credit card transactions are of paramount interest since, through the lens of purchases, they can provide a panoramic view of human life and represent the decisions made by individuals. These decisions and the underlying patterns behind them, not only affect the micro-economy, but also have direct influence on macro-economy [10]. However, unlike official economic indicators, credit card transactions do not readily provide insight into economy and need models that link individual spending activities to macro-indicators. Moreover, from the collapse of markets during the Great Depression to the failures that surrounded the global economic downturns of 2008, macroeconomics had failed to predict, prevent, or explain the occurrence of such momentous events. Much is due to the lack of the models in use, and the gap that exists in the models between individual behaviors and macro-phenomena.

In this paper, we aim to build connections between individual economic activities and macro-socioeconomic indicators on various spatial scales. Currently these indices are calculated on very coarse-grained spatial scales and reported very infrequently, e.g., quarterly or even annually. Our motivation is to be able to not only calculate indices in *real-time*, but also further zoom in to the city or neighborhood level providing people with indices that can describe socioeconomic characteristics of the exact location where they live, rather than provide them with average values that very often have large deviations. This information can be then used by policy makers, business investors or people deciding where to live [11].

Individual spending activities, which were investigated before the digital era, have been collected using field studies [12], questionnaires [13], surveys from users [14] and retailers [15]. The focus of those studies was mostly on finding correlations between demographic factors (e.g., age group, gender, education level, occupation or income) and either shopping patterns [14, 16, 17] or predisposition to use different payment methods such as bank cards, checks or money [18–21]. Since these studies were mostly based on survey results, they may have been affected by the fact that people could have altered their answers knowing that they were monitored. Today in this digital era in some cases information about people’s behavior is collected even without their awareness, let alone their informed consent. However, as bank card transaction data is highly sensitive and includes a lot of private information, access to it has been so far highly restricted. Therefore, related studies have been mostly focused on card systems [22–24], rather than on human behavior that can be derived from people using them. Nevertheless, a few studies focus on extracting some features of human behavior based on the credit card transit demostrate sactions to investigate how individual spending is affecting those individuals. For example, some studies wanted to uncover the predictability of people’s spending choices [25] or examine the relationship between wealth/income and financial mistakes [26]. In recent years, individual financial transaction datasets have been utilized to infer interesting perspectives on human mobility [27, 28], revealing different characteristics of people’s dynamics and spending habits with a novel scale-free classification of Spanish cities [29]. Moreover, financial transactions from retail market data is used to calculate a quantification of the average sophistication of satisfied needs of a population as a promising predictor of Gross Domestic Product (GDP) [30].

In this study, using individual credit card transactions, we design models to show how individual behaviors can be scaled up with varying degrees of resolution with the aim of uncovering macro-trends. Here, we perform a comparative quantitative analysis of city microeconomics, aiming to see how macroeconomic patterns could be understood starting from individual economic transactions. This is especially relevant as in the modern world not only businesses, but also entire neighborhoods, cities and regions compete for opportunities and investment resources. In this competition, it is very important to be able to quantify the current location’s success in comparison with the others and more importantly—the existing development potential. Bank data is unique for that purpose providing direct measurements for a number of aspects of human economic activity at a very fine-grained scale. Given the existing advanced real-time data infrastructure in the banking system, this creates a really exciting opportunity. Besides bank card transactions, recorded by Banco Bilbao Vizcaya Argentaria (BBVA), here we use Spain’s official statistical data on macro-socioeconomic indicators such GDP per capita, housing prices, unemployment rate, percentage of higher education, crime rate, and life expectancy on the province level (see Materials and methods for more information).

In the proposed predictive modeling framework, we aim to uncover different performance aspects of geographic areas at different spatial and temporal scales by designing and calculating different micro-socioeconomic indices solely based on individual bank card transactions. They comprise simple quantities, such as density of spending, fraction of foreign tourists in the area, along with more sophisticated ones, such as diversity of individual shopping patterns, business crossover through shared customers, and individual mobility (see S1 Table for more details). Second, we present our machine learning framework whose inputs are micro-socioeconomic indices and outputs are the prediction of official macro-socioeconomic indicators. Since the proposed predictive model is not specific to the region’s size, i.e., all the micro-socioeconomic indices can be evaluated at any scale, we can zoom in to more fine-grained spatial areas such as cities for which the official data does not exist. The scalability attribute of this model holds a great promise towards building a valid and consistent multi-scale measure of economic achievement and, more importantly, of existing opportunities across the territory. Understanding and accounting for the impact of the different parameters we used towards a global index of a location holds tremendous potential for more informed business decision making, more reliable urban planning, and more thoughtful policy making.

## Results

Through the lens of bank card transactions, we look at the economy and well-being of regions from three perspectives: 1) network of spending activities, where we investigate how regions are economically connected to each other and what are the boundaries, 2) micro-socioeconomic similarities across the boundaries, where we study similarities of regions based on micro-socioeconomic indices and 3) macro-socioeconomic indicators, where we build a predictive model that enable us to predict official statistics based on micro-socioeconomic indices. Although this analysis can be done at any geospatial scale, since provinces are the smallest region size with the reliable official statistics, we focus on 52 provinces and autonomous cities without loss of generality (see Materials and methods for more details about political divisions in Spain).

### Network of spending activities

**Fig 1:**
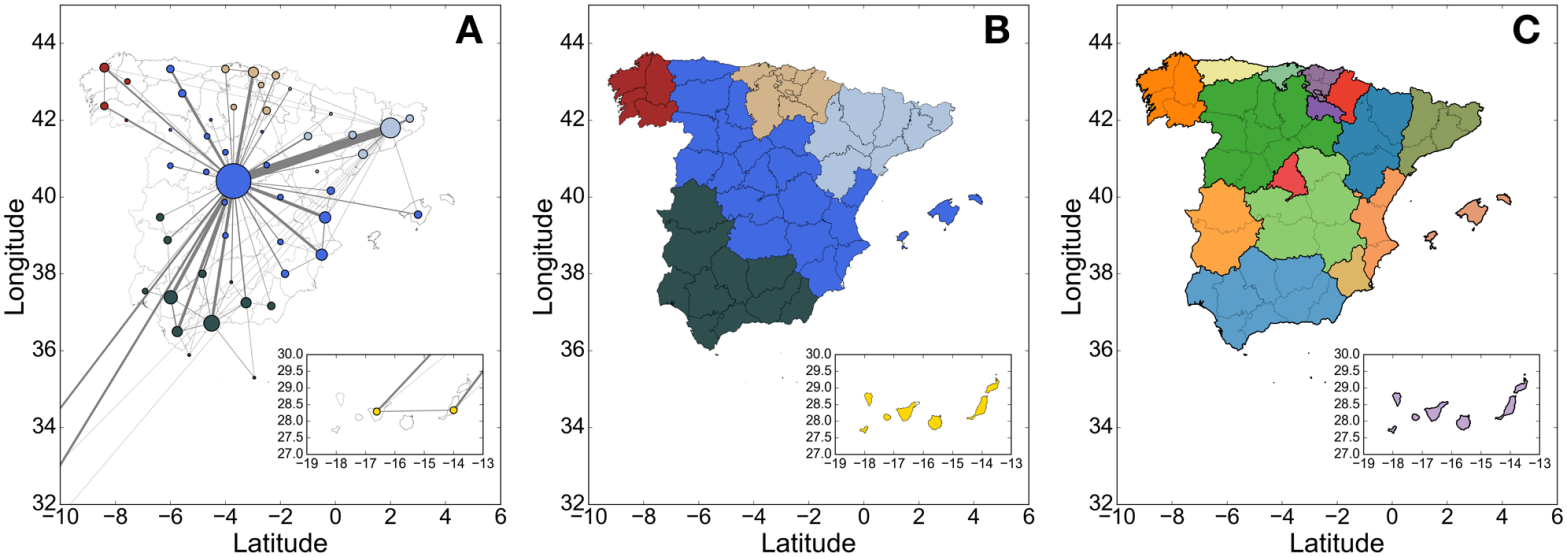
Network of the spending activity establishes links between regions. (A) A network of the spending activity in Spain: size of the nodes depends on the incoming activity (i.e., bank card activity from people of other provinces), and color of nodes reflects their community. For the visualization purpose, here we take into account only the most influencing links with more than 5.3 ⋅ 10^3^ transactions that correspond to the top 5% percentile. Namely, in this scenario we generate a network with 160 directed weighted links, where the weight corresponds to total amount of transactions from one province to another province. Visualization was done using *Gephi*. (B) The community detection algorithm, performed on the fully connected network described in the text and shown in S1 Fig, is able to identify 6 well-distinguished adjacent macro-communities. (C) Spain divided in 17 units called autonomous communities.

We build a network of economic connectivity of regions based on the amount of money spent by residents of a specific region at another region. In this way, the network has *N* = 52 nodes (i.e., provinces) and *E* = 2,652 directed links. A direct weighted link *l*_*ij*_ between two nodes/provinces *i* and *j* has been created if there was a transaction in the province *j* made by people living in the province *i*. The weight of the links *w*_*ij*_ corresponds to normalized total amount of transactions between the two provinces. In this scenario, the network consists of *E* ∼ *N*^2^ number of edges, which means that this is a fully connected graph (i.e., at least one transaction was made between any pair of two provinces). Fig 1(A) shows this network by visualizing only the top 5% of economic connections overlaid on a map of Spain. It shows the structure of the network as well as importance of each node based on its total amount of spending traffic.

In S1 Fig we report the normalized frequency distributions of the amount and number of transactions between Spanish provinces. It is clear that there is a correlation between the amount and the number of transactions as emphasized in S2 Fig.

Once the network is generated, we study the community structure of the spending activity network and compare it with the official division of Spain in 17 autonomous communities. We apply *Combo* algorithm [31] to detect the underlying macro-communities of this network. The algorithm partitions the network in 6 macro-communities as shown in Fig 1(B). Also the color of nodes in Fig 1(A) refers to the community they belong to. Fig 1(C) illustrates the official division of Spain in 17 autonomous communities, where each of them has its own Executive Power, Legislative Power and Judicial Power. Our modus operandi is able to detect 6 macro-communities, which in most of the cases are well aligned with the division of Spain in 17 autonomous communities, grouping autonomous communities together: the first one groups Andalucía and Extremadura together (dark green color) with the dominant provinces of Sevilla and Málaga, the second one covers Cataluña and Aragón (light blue color) with the dominant province of Barcelona, the third one is a big community that comprises Valencia, Murcia, Castilla-La Mancha, Madrid, Castilla y León and Asturias (dark blue color) with the dominant province of Madrid, the forth community is Galicia (red) with the dominant province of A Coruña, and the fifth one represents the Canary Islands (yellow). Finally, La Rioja, Navarra, País Vasco and Cantabria are all in the same community (brown) with the dominant province of Bizkaia. It is interesting that from the macro-communities shown by the network of spending, the cultural boundaries among provinces arise with the exception of the Mediterranean provinces of Castellón, Valencia, Alicante and Baleares that in principle have more in common with Cataluña than with Madrid from a cultural perspective. We use the so called Normalized Mutual Information (NMI) [32] (the code is available at https://sites.google.com/site/andrealancichinetti/software) in order to evaluate the goodness of the community detection. We compare the partition generated by Combo with the partition given by the 17 autonomous communities. The value of the NMI is 0.51 which is a high value if we consider that the partition generated by Combo is composed by 6 communities instead of the 17 autonomous communities used as testing benchmark: such a high value of the NMI means that this modus operandi allows to detect an important and significant geographical partition of the country.

### Socioeconomic similarities across the boundaries

Aggregated amount and number of transactions are important indicators of economic vitality of a region. However, they are not fully representative of underlying economic activities that lead to a complex macroeconomic behavior. Bank transactional big data provides an unprecedented opportunity to look at other aspects of human spending behavior. To this end, we have designed 33 micro-socioeconomic indices (see S1 Table) that can be evaluated at any spatiotemporal level. Among these indices, besides the ones representing microeconomic behavior of regions, there are indices characterizing social behavior of them. The correlation between these indices and official statistics is visualized in S3(A) Fig.

**Fig 2:**
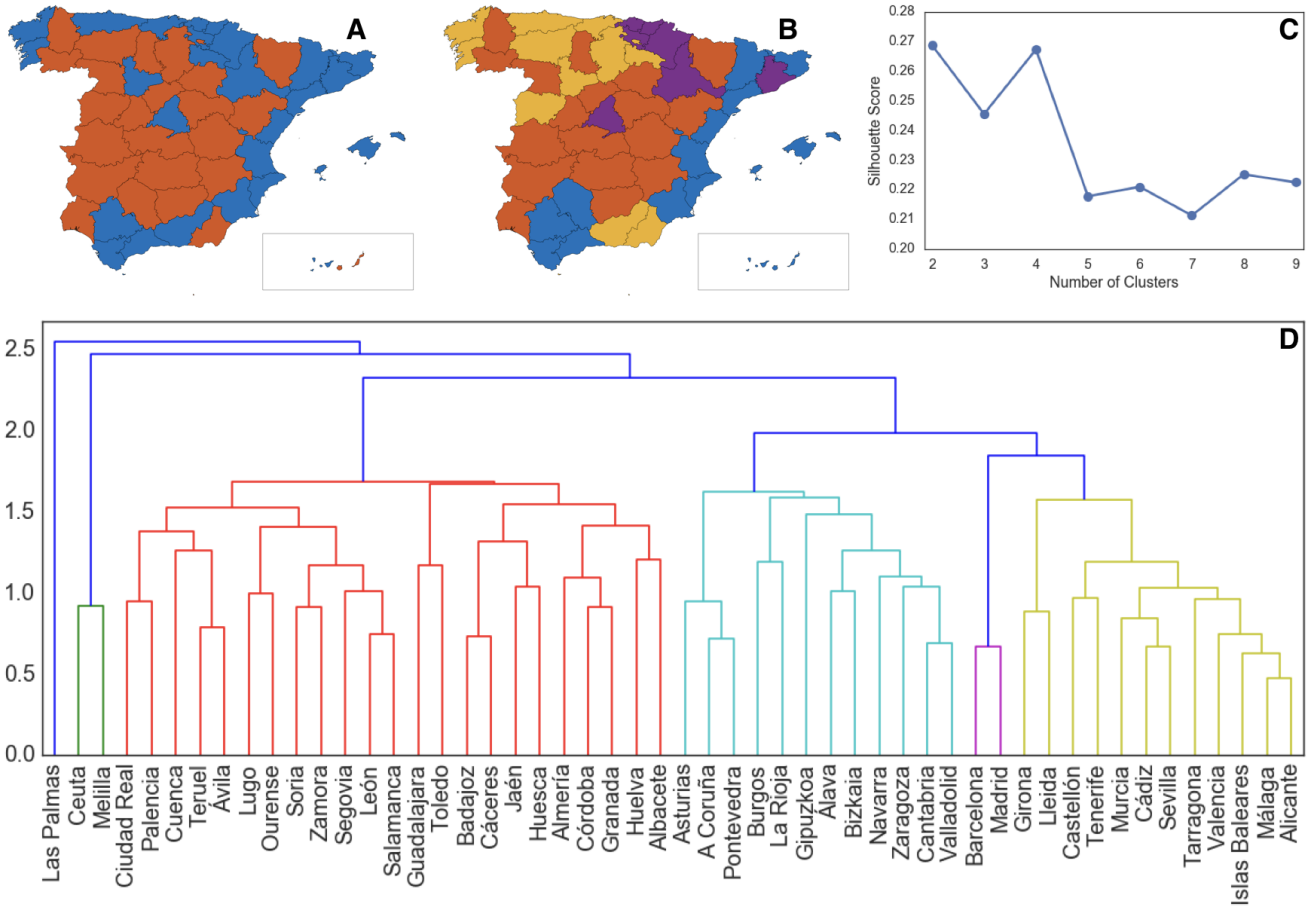
Cluster of provinces based on micro-socioeconomic indices. (A) *k*-means clustering with two clusters. (B) *k*-means clustering with four clusters. (C) The silhouette score for using different clusters in *k*-means clustering. (D) Dendrogram of agglomerative hierarchical clustering.

Using these micro-socioeconomic indices, we identify similarities between regions from two different standpoints: (1) partitioning dissimilar regions and (2) agglomerating similar regions. For the first one, we use *k*-means clustering on micro-socioeconomic indices with various numbers of clusters and evaluate them with Silhouette score (see Materials and methods for more details). Fig 2 shows the results of this clustering for two and four clusters, corresponding to the highest Silhouette scores. It is interesting that these clusters are not merely representative of economic performance of regions, but take into account some notion of social similarities. While the clustering based on official statistics, shown in S5(A) Fig, separates Spain into northern and southern parts, clustering based on micro-socioeconomic indices, as shown in Fig 2(A), utilizes a more complex similarity measure and separates the rural and less populated areas of the country (red) with median density of 26.8/*km*^2^ and median GDP per capita of 19,132 Euro), versus the more urbanized and more populated areas (blue, though Lleida is an exception in its cluster) with median density of 136.6/*km*^2^ and median GDP per capita of 22,597 Euro).

Clusters with more partitions also reveal new similarity patterns that are not aligned with the geopolitical representation of regions in Spain, as represented in Fig 2(B).

To look at similarities from the second standpoint, we employ agglomerative hierarchical clustering. Although both clustering methods try to define the relation between regions, this bottom-up approach is able to isolate very distinct regions. This hierarchy of clusters is represented as a dendrogram (a tree diagram), see Fig 2(D). The tree starts at the top with a unique cluster that gathers all the regions, and the leaves at the bottom are the clusters with only one region in each. By looking at this dendrogram, we can readily realize the abnormal behavior of Las Palmas, an island in Africa, and also Ceuta and Melilla, autonomous cities in the south of Spain and the north of Africa, with respect to the rest of Spain. It is interesting that this method makes a group consisting only of Madrid and Barcelona, which have a lot in common in terms of economies and tourism.

It is worth mentioning that clustering on micro-socioeconomic indices, constructed purely upon credit/debit card transactions reveals many patterns and much information about the performance of regions and their similarities that are not necessarily tied to their geolocations.

### Predicting official socioeconomic indicators

In this part, we are aiming to predict some of the most utilized official statistics merely based on information from credit/debit card transactions. These official statistics include some macroeconomic indicators like GDP per capita, housing prices, unemployment rate, as well as some social and wellbeing indicators like percentage of higher education, life expectancy and crime rate. As previously explained, we first build a feature space that consists of 33 parameters *x* = {*x*_1_, *x*_2_, …, *x*_33_}. Then we investigate the correlation between all pairs of micro-socioeconomic indices, as well as their correlation with official statistics to understand their relationship and if any of the features can be a sole predictor of any official indicator. S3(A) Fig shows a heat map of such a correlation matrix where we can observe linear correlation patterns. This figure illustrates intercorrelations among extracted micro-socioeconomic indices and suggests that all the conveyed information can be explained with less number of variables.

We address this redundancy by employing dimensionality reduction methods to automatically build a reduced features space. We apply Principal Component Analysis (PCA) on the micro-socioeconomic indices to obtain a new set of features that are linearly uncorrelated, called principal components (PCs) as shown in S3(B) Fig. One can observe in this figure that the first few PCs show a strong correlation with official statistics and these correlations fade away by going toward the last PCs. It demonstrates the fact that although we have the same number of PCs as the initial number of features, not all the PCs are equally important and many of them can be discarded without losing valuable information. S4 Fig shows that more than 89% of variability in the data can be represented by only 9 PCs, where the first PC alone is responsible for almost 40%. By removing linear correlations, through PCA, not only we get a clearer insight to the relationship of our features with official statistics, but we can also build a flexible model whose complexity can be tuned by choosing different number of PCs through cross validation iteration.

**Fig 3:**
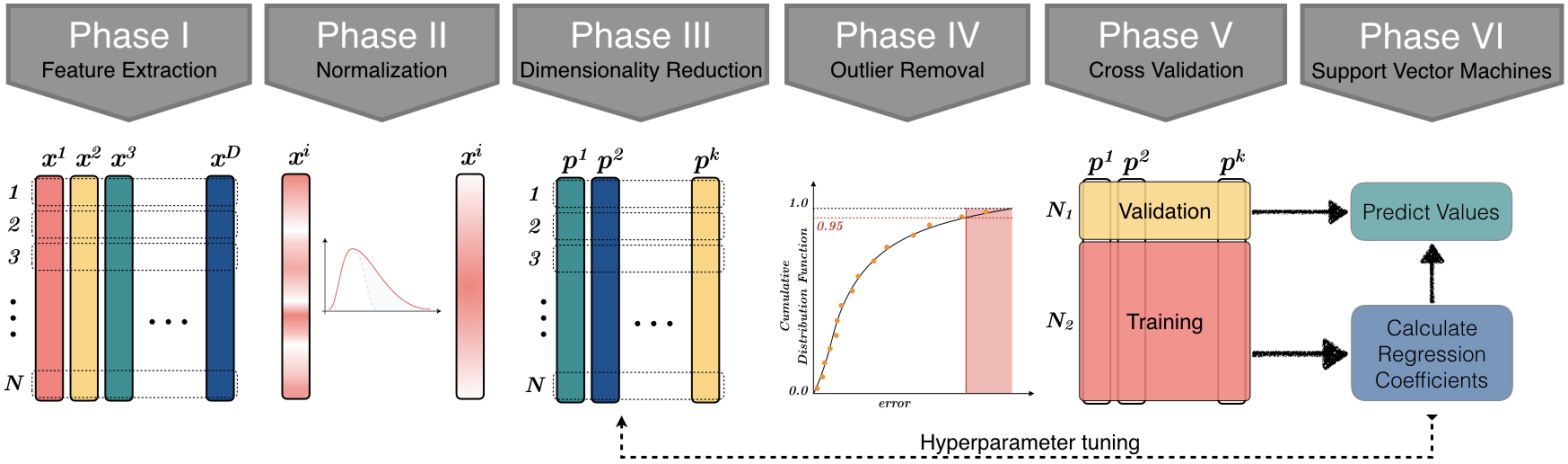
Modular machine learning workflow for predictive modeling of macro-socioeconomic indicators.

In this work, we propose a modular and flexible machine learning workflow for predictive modeling of official macro-socioeconomic indicators, which is illustrated in Fig 3 and explained in Materials and Methods.

We employ support vector machines (SVM) as a regressor at the core of our methodology, which offers the best performance in prediction of all six official indices. Table 1 lists for each official index the optimum number of PCs needed to maximize the prediction quality (*R*^2^). An interesting observation here is that each target index requires different level of complexity and information to be able to provide a decent prediction, e.g., while housing price level and unemployment rate are explainable using only the first two PCs, GDP per capita require more information from seven PCs.

**Table 1:** Predictive modeling of official socioeconomic indicators based on bank big data. The prediction coefficient of determination (*R*^2^) and optimal number of required PCs for predicting each macro-socioeconomic indicator are reported.

Quantity	Validation *R*^2^	Number of PCs
GDP Per Capita	0.729	7
Housing	0.764	2
Unemployment	0.738	2
Education	0.753	5
Crime	0.620	8
Life	0.558	5

**Fig 4:**
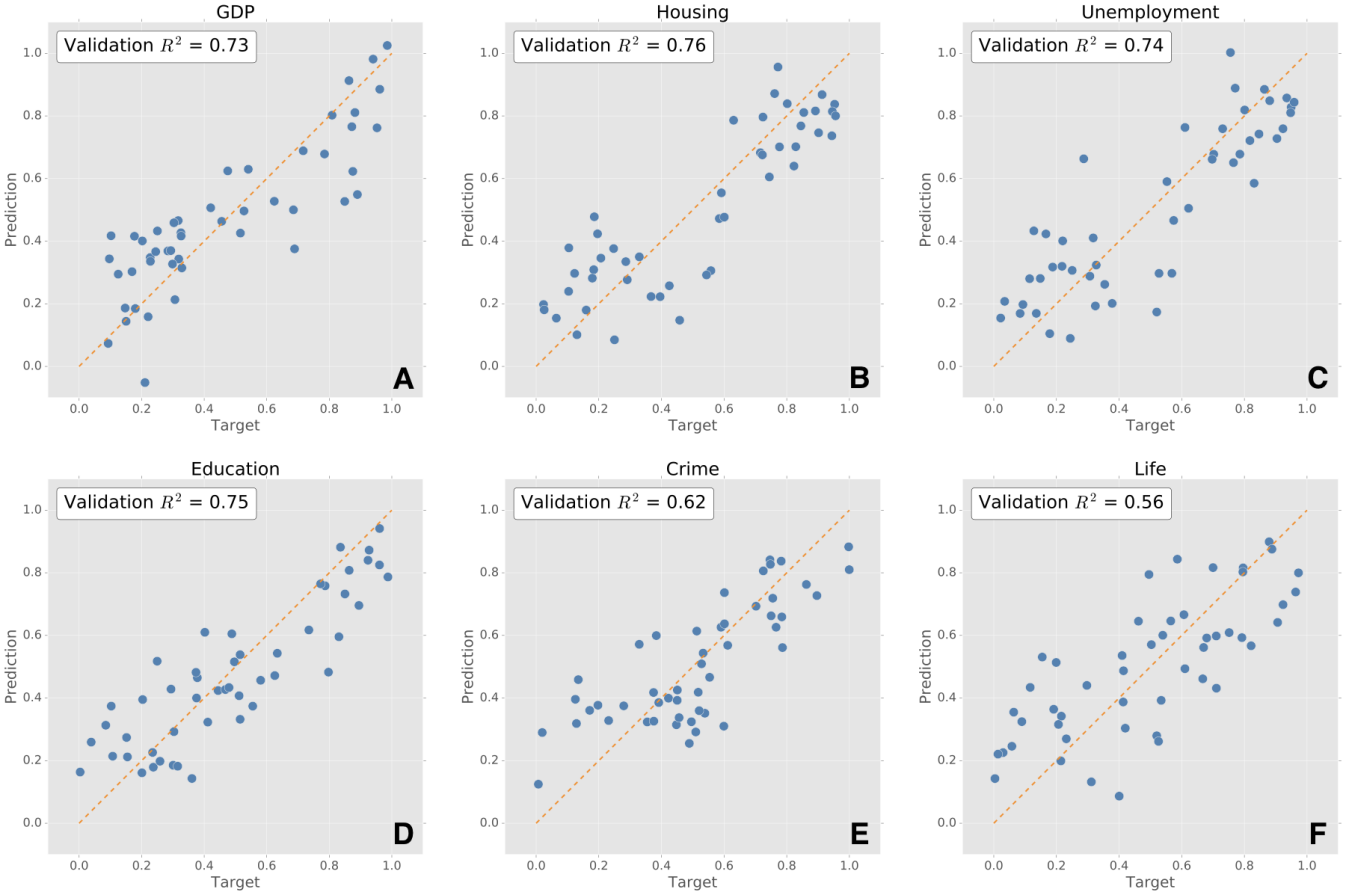
Outcome of predictive modeling of official socioeconomic indicators based on individual transactions. Each subplot shows the performance of the proposed methodology to predict six official socio-economic features (target). The prediction is based on commercial indices built upon financial transactions and employing an iterative methodology, which takes advantage of PCA, support vector machines. The orange dotted lines indicate the ideal prediction.

Fig 4 compares the predicted value of official indices with the corresponding expected values. It shows that our predictive model performs decently on macroeconomic indicators such as GDP per capita, housing price level and unemployment rate, which have explicit relation to people’s spending. The proposed methodology also demonstrates an acceptable performance in predicting indicators with not straightforward relationship with spending behavior of people, such as percentage of higher education. For the more social and wellbeing indices, with moderate prediction *R*^2^ values, although it nicely captures the general trends of regions, it urges for more information from different aspects of people’s life in order to obtain a proper prediction power.

**Fig 5:**
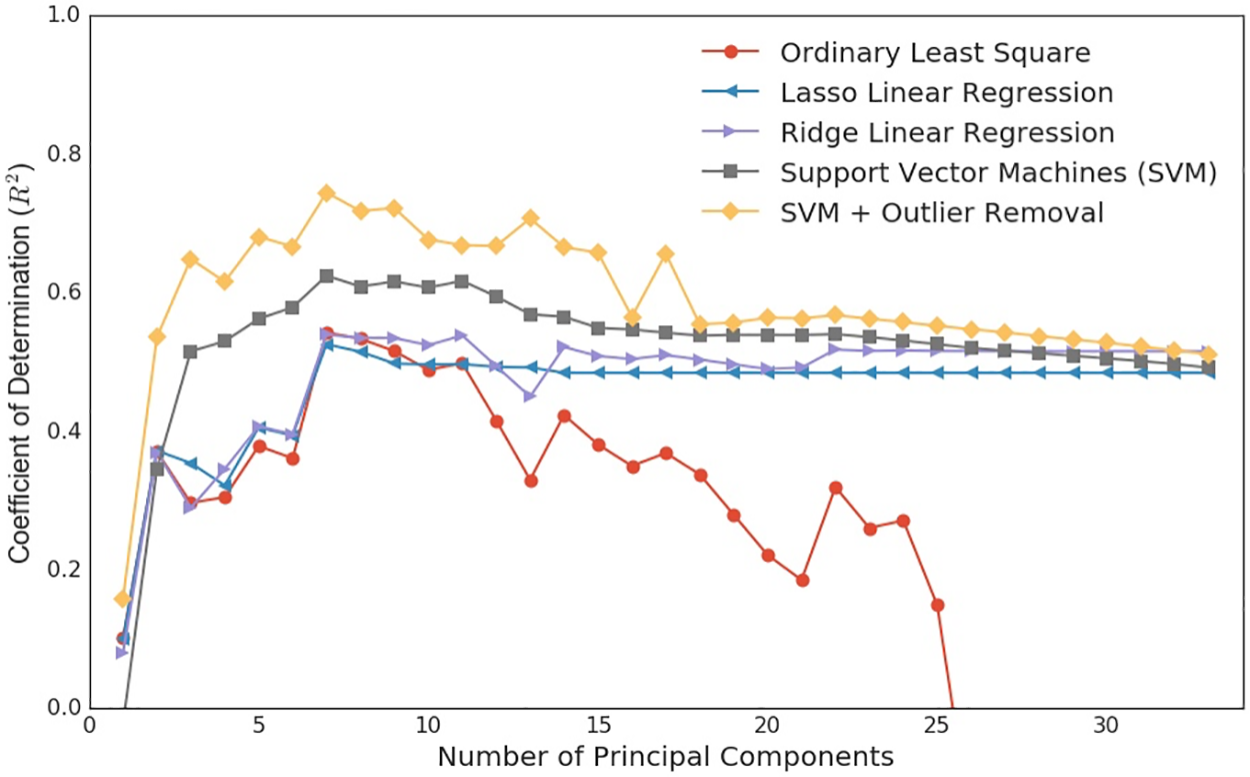
Comparison of performance of proposed methodology using different regression methods. The proposed method can utilizes any regression method at its core and here its performance using four common regression methods on predicting GDP per capita based on micro-socioeconomic indices is illustrated. The rightmost points can be interpreted as the values without using dimensionality reduction phase.

This methodology shares the concept of regularization and feature selection with regularized regression methods, such as Lasso and Ridge (see Materials and methods section) which improve interpretability of the statistical models and prediction accuracy. However, it provides a general framework to equip any regression method with a such attribute. To verify this concept we compare the proposed methodology with different regularized methods. Fig 5 shows the prediction performance of various regression methods, such as ordinary least square (OLS), Lasso linear regression, Ridge linear regression and SVM. The performance is evaluated in term of *R*^2^ values and employing K-Fold cross validation (see Materials and methods). We also calculate their p-value in order to evaluate the significancy of each regression and accounted for Bonferroni correction, see S6 Fig for significancy of each method on GDP per capita prediction.

Although here we use the aforementioned dimensionality reduction method, the rightmost value of this plot, which means using all the PCs without reduction, is essentially equivalent to using the regression methods without adopting the PCA phase. This figure can bring us to three important observations. First, OLS method completely failed at predicting targets with larger number of PCs since it does not have the regularization ability. Second, the regularized linear regression methods significantly improve the prediction accuracy and are able to provide a proper *R*^2^ values similar to the nonlinear method (SVM) with high number of features. Third, adopting the PCA phase improves all the regression method, even methods with regularization, and help a simple method without the regularization mechanism (e.g., OLS) performs decently. Moreover, due to the huge difference in geopolitical situation of provinces, they might show anomalous behaviors in official statistics and hinder the prediction. To alleviate this issue we add the outlier removal phase, explained in Materials and Methods section. As illustrated in Fig 5 for SVM method, it demonstrates a considerable improvement in the predictive model.

## Discussion

Due to increase of the number and complexity of interconnected systems, systems today do not only interact through conventional interfaces, explicitly defined at design-time, but also through the physical world including humans [33]. Not only can we not control outcomes of artificial system designs, but also impacts of policy makers’ decisions may have unexpected and undesirable consequences on our societies. Some of the negative consequences could be stopped and reversed if we had real-time information about the outcomes of our policies or design choices. In the cases of artificially designed systems, information we collect often is real-time, unlike in the cases of policies affecting our societies which often only rely on data officially collected quarterly, yearly or rarer. On the other hand, since the advent of pervasive digital technology in our everyday life, people are leaving an increasing number of digital traces. Creating data analytics and data visualization from this new layer of information sheds light on human behavior from the micro-scale of individuals and households, which can scale up to a macro-scale characterization of cities and countries and can provide more frequent feedback on how our policies are working.

In this paper, we present a framework that allows decision makers to see almost immediately what the impacts of their socioeconomic measures are. Namely, the vast number of bank card transactions each day carries a collective knowledge about the society and its economy. As people are using more and more debit and credit cards to purchase their goods and services, information about individual’s purchases becomes more available and the analyses reflect more accurately the underlying social and economic behavior of people. Due to sensitivity of such data, many anonymization techniques are used to remove identifiable information and protect individual privacy although recent studies warn about re-identifiability of people based on their unique behavior [34, 35].

Here, we explore millions of anonymized credit/debit card transactions in Spain, provided by BBVA, which holds a ubiquitous banking infrastructure in the country. The data provides an opportunity to uncover macro-trends derived from a fine-grained scale of individual economic behavior. In light of the failure of past decades to produce models that effectively predict and explain the macroeconomic trends, we noticed that a gap exists between models of micro-behaviors and macro-phenomena.

In our framework, we extract 33 different characteristics of socioeconomic behavior quantifiable from the dataset of BBVA bank card transactions, and then evaluate them on the example of Spain. We show that those quantities could be used for estimating economic performance of the regions in the country, as the proposed supervised machine learning technique performs well on the validation samples for predicting major official statistical quantities such as GDP per capita, housing prices, unemployment rate, level of higher education, life expectancy and crime rate at the scale of Spanish provinces. Building the connection between individual transactions and official indicators, reported at specific time points, enables us to predict these indicators at arbitrary time points and also to evaluate temporal variation of economic performance of the regions, which is especially useful since official statistics are more static and cannot give a really fine-grained longitudinal perspective. Besides spanning the time domain, this data-driven approach allows us not only to change the spatial scale to evaluate indicators in the spatial scales that no official statistics is available, but also to draw arbitrary boundaries that are not commensurate with official boundaries of regions and evaluate corresponding macro-indicators. The ability of this machine learning platform to be generalized over time and space set the ground for adaptive policy making approaches in which a policy can be adjusted in time and be customized for specific regions.

The generalization of the proposed framework fairly relies on the availability of target information, particularly in determining the number of PCs in dimensionality reductions part. In a broad range of applications, when the target information is available, like official statistics at province level, we can easily find the optimal hyper parameters by maximizing the prediction accuracy. However, it is not the case in many real-world applications where the target information (ground truth information) is partially available or not available at all. We can cope with these scenarios in different ways depending on the type and availability of data. When the target information can be achieved through other sources for a small subsample of data despite the fact that such information is not feasible to acquire for the whole dataset, or when the target information is available at a spatial scale (e.g., provinces), but not available at a finer spatial scale (e.g., cities), we can use the transferability of these models with respect to sample size and spatial scale. In this way, the model is trained on the smaller or coarser dataset with ground truth information and then the learnt parameters and hyper-parameters (e.g., number of PC’s) are used for prediction over larger or spatially finer datasets. However, it is not plausible when there is not any target information available at all. In this case, we can eliminate any supervised part of the methodology with their unsupervised approximation. Hence, we can select the number of PCs based on a cut-off in the PCA’s explained variable spectrum (e.g., 90% cumulative variability). Although in this way the number of PCs is not optimal with respect to the target indicators, they can efficiently help in overcoming the inter-correlation of feature space and reduce overfitting while keeping the most important features.

As we mentioned, the introduced micro-socioeconomic indices are scale independent and thus can be evaluated for any spatial division; however, evaluating such indicators for building a reliable predictive model in practice is a two-fold problem. Due to the limited number of transactions and heterogeneous market share of BBVA, in the finer scale, there would not be enough information for a robust calculation of the micro-socioeconomic indices. Hence, by coarsening the scale, we can calculate more reliably these indices. On the other hand, at the very coarse scales, where the number of regions is rather small, we will end up with a few data point to build the predictive model. This fact not only affects the robustness of regression models, but also causes the more complex nonlinear models to overfit the training set and consequently aggravate the validation score.

The proposed approach in this paper holds tremendous potential in its far-reaching applicability to discover patterns that can be used in urban planning, policy-making and business decisions. We believe that the tools similar to the one we designed and deployed in interactive web application (https://urban-lens.herokuapp.com) will also enable citizens to (1) obtain official statistics of provinces as well as spending characteristics, (2) visualize each of them through a density plot over the country, and (3) compare the provinces based on commercial indicators and official statistics, quantitatively and visually.

## Materials and methods

### Geographical information

Our study region is Spain with an area of 505,519 *km*^2^ and 46,507,760 of inhabitants (2014). It is bordered to the northeast with France (which is separated from the chain of the Pyrenees) and Andorra, on the south by the Mediterranean Sea and Gibraltar (small possession of the United Kingdom) and, in Africa, with Morocco (through the autonomous cities of Ceuta and Melilla, its exclave). Spain is divided into 17 autonomous communities (comunidades autónomas) which are further divided into 50 provinces, plus 2 autonomous cities: Ceuta and Melilla (officially designated as Plazas de Soberanía en el Norte de África). Ceuta, Melilla and other small islands, which extend over 0.65 *km*^2^ and count 312 inhabitants are the remains of the vast colonial empire that the country possessed. In total, Spain has 31.65 *km*^2^ of territory in North Africa, populated by 138,228 inhabitants.

### Bank card transactions dataset

We analyze Spain microeconomic activity during the year 2011 represented by the complete set of bank card transactions recorded by BBVA during 2011, all over Spain (i.e., 50 provinces plus 2 autonomous cities: Ceuta and Melilla). All the aggregated data used in this paper at the province level are available to public at http://senseable.mit.edu/urban-lens/ although access to individual transactions is protected by a non-disclosure agreement and is not publicly available. Transactions stored in our dataset were performed by two groups of bank card users. The first one consists of BBVA direct customers, residents of Spain, who hold a debit or credit card issued by BBVA. In 2011, the total number of active customers was around 4.5 million, altogether they executed more than 178 million transactions in over 1.2 million points of sale, spending over 10 billion euros. The second group of bank card users includes over 8.6 million foreign customers of all other banks abroad coming from 175 countries, who made purchases through one of the approximately 300 thousand BBVA card terminals. In total, they executed additional 17 million transactions, spending over 1.5 billion euro. Due to the sensitive nature of bank data, our dataset was anonymized by BBVA prior to sharing, in accordance to all local privacy protection laws and regulations. As a result, customers are identified by randomly generated IDs, connected with certain demographic characteristics and an indication of a residence location—at the level of zip code for direct customers of BBVA and country of residence for all others. Each transaction is denoted with its value, a time stamp, a retail location where it was performed, and a business category it belongs to. The business classification includes 76 categories, which were further aggregated into 12 meaningful major groups (e.g., purchases of food, fashion, home appliances or travel activities).

### Micro-socioeconomic indices

In this first step of our model we built a feature space using the BBVA dataset of individual spending behavior for the period of one year and by extracting 33 different microeconomics indicators that explain economic behaviors from both customer and business sides (see S1 Table). Before calculating the aforementioned parameters, the BBVA dataset had to be pre-processed in order to compensate for potential bias introduced by the spatial inhomogeneity of BBVA market share. The first concern was: what is BBVA penetration in the whole banking market for the given area (i.e., what is the ratio of BBVA customers and economically active population)? Therefore, in order to estimate the total domestic customer spending volume, customers’ activity was normalized by the bank market share corresponding to their residence location and grouped at the level of provinces. Another type of bias is related to unequal distribution of foreign customers performing transactions in BBVA point of sale terminals in different locations across the whole Spain. In this case the normalization procedure relied on BBVA business market share defined, for the purpose of this study, as a ratio of bank card transactions executed by domestic customers in BBVA terminals and their transactions in all other terminals located in the considered area. The appropriate normalization allows estimation of the total spending volume of foreign customers visiting a particular location.

The full list of indicators at the province scale, available in S1 Table, can be split in three macro-categories: (i) customer and (ii) business (i.e., merchant) oriented and (iii) categories of spending. The first eleven indicators refer to the economic activity inside each province. Indicator 1 has been computed by evaluating the average density of number of transactions made within 1 *km*^2^ of the province area, while Indicator 2 refers to the average density of amount of money spent, and Indicator 3 denotes the ratio between total amount and number of transactions made by all customers within the considered area. Indicators 4, 5 and 6 are more focused on the customer side. Indicator 4 evaluates the average number of transactions per customer, i.e., the ratio between the total number of transactions made by residents of the area and the number of active residents in terms of transaction activity. Indicator 5 computes the fraction between the total amount and the number of transactions made by residents of the considered area everywhere in the country, while Indicator 6 evaluates the percentage of the number of transactions made within the area by its domestic out-of-province visitors. Moreover, we also evaluated the effect of the foreign activity by considering the percentage of the number of transactions made within the area by its foreign visitors.

In order to also include the effect of the structure of activity by its type, we consider something that what we call—earning and spending *diversity*. In that sense, Indicators 8 and 9 represent transactions made over the number of top business categories (of 76) enough to cover 80% of the total activity of area residents or activity within the area, respectively. Additionally, Indicator 10 reflects the number of active businesses within the area per *km*^2^. Furthermore, Indicators 11 to 21 correspond to the specific properties of the structure of spending activity within the area taking into account spending in different business categories, such as food, taxi, public transportation, etc. Finally, we evaluate the effect of the temporal activity by distinguishing nighttime and weekend temporal windows. For the purpose of defining Indicators 22 to 29 we assume that nighttime activity happens between 10 PM and 6 AM, while weekend activity counts for transactions made on Saturdays and Sundays. Indicators 30 to 32 reflect the customer activity inside or outside their provinces. The last indicator computes the percentage of the total amount of transactions made by residents in the *expensive* businesses, i.e., those which average transaction amount is above average for the corresponding business category.

### Dataset of official statistics

Many official statistical quantities are available for Spanish province level, being included in official Spanish statistic reports from Instituto Nacional de Estadística and Eurostat web pages. We also consider statistics on customer wealth and housing categories available on the census unit level (over 32,000 units), which allow aggregation to the municipality (over 8,000), comarca (368) and provinces and autonomous cities (52) scale. We perform aggregation to the bigger spatial units computing weighted averages of the considered parameters (weighting each census unit contribution according to its population). On that end we compute an estimate for average income, percentage of high/low income residents, percentage of individual and new houses. As mentioned in the Introduction, a huge number of indicators can be used to characterize quality of life for whole countries and their citizens. In this work we decided to focus on six macro-socioeconomic indicators for the year 2011 that are available for Spanish province level and that are included in official Spanish statistic reports from websites of Instituto Nacional de Estadística (http://www.ine.es) and Eurostat (http://ec.europa.eu/eurostat): GDP per capita, housing price level, unemployment rate, crime rate, percentage of higher education, and life expectancy.

We choose GDP per capita as it is widely used as a benchmark of successful public policy initiatives and as the primary objective of the lending decisions of major global economic institutions. The advantage of GDP is that it measures the aggregate economic activity within a country, but the downside is that economic activity generated for whatever purpose (e.g., building prisons or schools, spending more on health care, whether or not it is medically beneficial) raises GDP per capita in the same way. In addition to economic indices, we also use social ones that are compiled by the Statistics Division, Department of Economic and Social Affairs of the United Nations Secretariat (http://www.un.org/en/development/desa/index.html) using many different national and international sources. Namely, the indices presented in this paper consist mainly of the minimum list that has been proposed for follow-up and monitoring implementation of major United Nations conferences on children, population and development, social development and women. This minimum list is contained in the Report of the Expert Group on the Statistical Implications of Recent Major United Nations Conferences (E/CN.3/AC.1/1996/R.4). Technical background on the development of social indices is available in two United Nations publications: *Handbook on Social Indicators (United Nations publication, Series F, No. 49, 1989)* and *Towards a System of Social and Demographic Statistics (United Nations publication, Series F, No. 18, 1975)*. All aforementioned indices are provided for the following areas: population, health, housing, education and work.

### Machine learning workflow

The proposed machine learning workflow, from the raw data to the prediction phase, is demonstrated in Fig 3. The first step in building our model is to normalize all micro- and macroeconomic parameters to be between 0 and 1 by fitting an appropriate distribution, followed by performing dimensionality reduction using standard PCA. After doing PCA, the next step in building of our model process is to train the model using the selected feature space that explains the statistical quantities at the considered spatial scale. The proposed machine learning workflow is modular and flexible and allows us to use the regression methods of our choice.

#### Normalization

Each of the devised predictive indicators (S1 Table) and socioeconomic features has different scales, and thus we need to normalize and transform them to a common basis. In order to do that and also to reduce the influence of outliers (extreme values) in the data without removing them, we use sigmoidal normalization. In doing so, we transform the data using cumulative distribution function of fitted distribution into quantile space. This is similar to the quantile normalization introduced in [36], but instead of using the certain empirical distribution, in this paper we use the actual best-fit distribution function. For each indicator and feature we find distribution parametrized by *θ*, which minimizes negative log likelihood estimation:

$$\begin{array}{c} -\log L\left(\theta |x\right)=-\sum_{i=1}^{N}\log f\left(x_{i}|\theta \right) \end{array}$$ (1)

where L is likelihood function and *f* is viewed as the probability of *x*_*i*_ sampled from a distribution parametrized by *θ*.

#### Clustering

In this paper, we perform cluster analysis of the regions with two fundamentally different method: *k*-means clustering, and agglomerative hierarchical clustering.

In *k-means clustering*, we start with some random centroids whose Voronoi cells define clusters and continue to optimize their position. This algorithm aims to partition regions in *k* clusters (*c*_*i*_) by minimizing within-cluster sum of squares,

$$\begin{array}{c} \min\sum_{i=1}^{k}\sum_{x\in c_{i}}{∥x-\mu_{i}∥}^{2}, \end{array}$$ (2)

where *μ*_*j*_ is the centroid of cluster *i*. In general, this metric is not normalized and poses problem in high-dimensional spaces, where Euclidean distances tend to become inflated due to the curse of dimensionality. To ameliorate this problem, we use PCA prior to performing clustering. Moreover, since the outcome of *k*-means can highly depends on the initial choice of centroids, we run the clustering with 1000 times with different initializations using the *k-means* *++* [37] for smarter selection of centroids that speed-up the convergence.

To evaluate the performance of clustering we use *Silhouette score*, which is a measure of how similar a region is to its own cluster compared to regions from other clusters. For each sample, the Silhouette coefficient is defined as follow,

$$\begin{array}{c} s\left(i\right)=\frac{b(i)-a(i)}{max(a(i),b(i))} \end{array}$$ (3)

where *a*(*i*) is a measure of dissimilarity of *x*_*i*_ within the cluster (the mean distance to all other points in the same cluster) and *b*(*i*) is a measure of lowest average dissimilarity of *x*_*i*_ to any other cluster (the mean distance to all other points in the neighboring cluster). Then we use mean of all Silhouette coefficients to evaluate Silhouette score for the clustering.

On the other hand, the *agglomerative hierarchical clustering* takes a bottom-up approach, where we start with one cluster for each region and as we move up the hierarchy, the pairs of clusters are successively merged based on their similarities. The clusters can be merged together based on different linkage criteria. Here we used *average linkage* which is the average of the distances between all observations of pairs of clusters.

#### Principal component analysis

Since many of the indicators are strongly correlated with each other (please refer to S3 Fig), the next step is to perform dimensionality reduction using standard PCA [38]. PCA can be used to compress the information from a large number of variables to a smaller dataset while minimizing the information lost during this process [39]. PCA seeks four goals: [40]

1. extract the most important information from the data;
2. compress the size of the dataset by keeping only the important information;
3. simplify the description of the dataset; and
4. analyze the structure of the observations and the variables.

PCA finds a set of linearly uncorrelated basis by applying either Singular Value Decomposition (SVD) of the indicators or eigenvalue decomposition of the covariance matrix of indicators. Considering the following covariance matrix,

$$\begin{array}{lll} X & = & \left[\begin{array}{lll} — & x_{1}^{T} & —\\ — & x_{2}^{T} & —\\ & \vdots & \\ — & x_{n}^{T} & — \end{array}\right]\\ C & = & {\left(X-\bar{X}\right)}^{T}\left(X-\bar{X}\right) \end{array}$$

where $\bar{X}$ is a vector of the average of *X* at each dimension, each set of eigenvector ($p_{i}\in R^{D}$) and eigenvalue (λ_*i*_) satisfies:

$$\begin{array}{c} Cp_{i}=\lambda_{i}p_{i}. \end{array}$$ (4)

Each eigenvector is called a PC, whose corresponding eigenvalue defines its importance or amount of explained variability. Thus, by sorting eigenvalues from largest to smallest, we can select first *d* corresponding PCs and define a reduced basis for projection, which is an optimal basis that minimizes the projection error:

$$\begin{array}{ccc} P & = & \left[p_{1}^{T},p_{2}^{T},\cdots ,p_{d}^{T}\right]\\ z_{i} & = & x_{i}P \end{array}$$

where $z_{i}\in R^{d}$ is the projection of $x_{i}\in R^{D}$ and *d* ≪ *D*.

It provides us with a set of Eigenvectors, called PCs, and associated Eigenvalues, which represent the amount of explained variability. Cumulative sum of this sorted Eigenvectors gives the notion of how many PCs is required to recover a desired variability of the system. S4 Fig shows the amount of explained variability as a function of preserving number of PCs. This can also be interpreted as a tuning parameter to define the level of details in the reduced description of the system. In this research, we analyze the performance of the models by using different number of PCs and pick the one that maximize the model performance.

Selected PCs are then used as a feature space for training our model to predict quality of life parameters at the province level in Spain. As mentioned before, this model can be further applied for predicting quality of life parameters on much more fine-grained spatial scales (e.g., cities, districts and smaller neighborhoods) for which consistent official statistics does not exist.

#### Outlier removal

In regression analysis, an outlier is a data point that by far does not follow the general trends in the dataset. It may have different sources, such as excessive variability in the measurement and recording error. There are many possible way of finding outliers [41]. Here, we evaluate for each data point its out-of-sample error, using leave-one-out cross validation. In this way, we will have the distribution of out-of-sample errors, which shows how much each data point is not following general trend of the rest of data. Then, we fit a lognormal distribution function to find the error distribution and use the 95 percentiles of this distribution as a cutoff for removing outliers.

#### Cross validation

Learning the parameters of a predictive model, we need to test them on a set of new data from which we have not learned the parameters (unseen data). In order to do that we need to hold out part of the available data and try to build the model based on the rest of the data, called *training set*. Then we use the hold-out data, *test set*, to evaluate the goodness of prediction with some metrics. This procedure called *cross validation*. There is a variety of cross validation methods, such as *K*-Fold and leave-one-out method, and random permutation. In this paper, we employed *K*-Fold method, where we divide the samples into *K* equal groups (folds) and at each iteration we put aside one of these groups as *test* set and consider the rest as *training* set. Thus, at the end we will have prediction for all the samples since each sample has been in the *test* set exactly once. Here we use *K* = 10.

#### Regression methods

After doing PCA, the next step in building of our model process is to train the model using the selected feature space that explains the statistical quantities at the considered spatial scale. We denote the predictors with an *n* × *d* matrix *X*, where *n* is number of regions and *d* is the number of variables that is taken into account, and each target with a vector *y*.

*Linear Regression* We start with Ordinary Least Square (OLS), in which a coefficient is calculated for each predictor as well as an additional one for the perception. However, this method is prone to overfitting. To overcome this issue, we employ penalized regression, also known as regularized regression, in which a penalty is added for over-confidence in the parameter values. Two types of penalties are typically used for regression: L1 penalties, known as *Lasso* regression, and L2 penalty, known as *Ridge* regression.

$$\begin{array}{cc} \underset{\beta }{\arg\min}\;∥y-\beta^{T}X∥ & \;\;\;(\text{Ordinary})\\ \underset{\beta }{\arg\min}\;∥y-\beta^{T}X∥ & +\alpha_{1}\sum_{i}\left|\beta_{i}\right|\;(\text{Lasso})\\ \underset{\beta }{\arg\min}\;∥y-\beta^{T}X∥ & +\alpha_{2}\sum_{i}\beta_{i}^{2}\;(\text{Ridge}) \end{array}$$

*Support Vector Regression* We implement Support Vector Machine (SVM) [42] with radial-basis-function kernel. This method implicitly maps the inputs into high-dimensional feature spaces using the kernel trick and then try to build the regression model upon it.

The error function, which gives zero error if the absolute difference between the prediction $\hat{y}\left(x\right)$ and the target *y*(*x*) is less than *ϵ* where *ϵ* > 0, is given by:

$$\begin{array}{c} E_{\epsilon }\left(\hat{y}\left(x\right)-y\left(x\right)\right)=\{\begin{array}{cc} 0, & \text{if}\;\;|\hat{y}\left(x\right)-y\left(x\right)|<\epsilon \\ |\hat{y}\left(x\right)-y\left(x\right)|-\epsilon , & \text{otherwise} \end{array} \end{array}$$ (5)

We therefore minimize a regularized error function as following,

$$\begin{array}{c} C\sum_{i=1}^{N}E_{\epsilon }\left({\hat{y}}_{i}-y_{i}\right)+\frac{1}{2}{∥w∥}^{2}, \end{array}$$ (6)

where *C* is the regularization constant. In this work, we use SVR implementation of Scikit-learn [43] Python packages. See Smola and Schölkopf [44] for more details on support vector regression.

To determine the degree to which the model fits our data, we use the coefficient of determination (*R*^2^) metric, i.e., measure based on unweighted residual sums of squares. The benchmark is the residual sum of squares in the intercept-only model, with fitted mean $\bar{y}$. In this paper, we use the (unweighted) residual sum of squares yield as:

$$R^{2}=1-\frac{\sum_{i=1}^{N}{\left(y_{i}-\hat{y_{i}}\right)}^{2}}{\sum_{i=1}^{N}{\left(y_{i}-\bar{y_{i}}\right)}^{2}},$$ (7)

where $\hat{y_{i}}$ is the predicted value by the machine learning algorithm and *y*_*i*_ is the *target* value.

## Supporting information

S1 Table Micro-socioeconomic indices consist of 33 indices representing economic and social behavior of residence and businesses. These indices can be evaluated at various spatial scales from small scale of zip codes to larger scale of provinces and countries based on the bank card transactions.
(PDF)

S1 Fig Frequency of inter-regional transactions. Normalized frequency distribution for the total amount (A) and total number of transactions (B) between provinces in Spain.
(TIF)

S2 Fig Number of transactions vs. amount of transaction. The relationship between total amount and total number of transactions can be describe with a power law although the exponent is close to one (0.96).
(TIF)

S3 Fig Correlation matrix of the micro-socioeconomic indices and official indicators. The correlation matrix is calculated for the socioeconomic indices before (A) and after (B) applying PCA (without reducing the dimensionality of data).
(TIF)

S4 Fig Explained variability in PCA. The cumulative sum of the ordered eigenvalues associated with the PCs of micro-socioeconomic indices is illustrated.
(TIF)

S5 Fig Clustering based on official statistics. *k*-means clustering of Spanish provinces based on official socioeconomic indicators considering two clusters (A) and four clusters (B).
(TIF)

S6 Fig P-value of prediction of different regression methods when using various number of PCs. The dotted green line shows the significant level (*α* = 0.0003) when applying Bonferroni correction with family-wise error rate *α*_*F*_ = 0.05 and number of comparison *m* = 33 × 5. The bottom figure is zoomed in to the smaller values.
(TIF)
