# Supplementary material for: Socioeconomic characterization of regions through the lens of individual financial transactions
Source: PLoS One. 2017 Nov 30;12(11):e0187031. doi: 10.1371/journal.pone.0187031 (PMC5708635; doi:10.1371/journal.pone.0187031)
Supplement: S1 Table — These indices can be evaluated at various spatial scales from small scale of zip codes to larger scale of provinces and countries based on the bank card transactions. (PDF) [file pone.0187031.s001.pdf]

Table S1: **Micro-socioeconomic indices** consist of 33 indices representing economic and social behavior of residence and businesses. These indices can be evaluated at various spatial scales from small scale of zip codes to larges scale of provinces and countries based on the bank card transactions

Index	Description
1	Density of the spending within the area
2	Density of the earnings within the area
3	Average amount of a single transaction earned within the area
4	Annual number of transactions per customer living in the area
5	Amount of transaction performed by customers living within the area
6	Percentage of area activity, received from out-of-province visitors
7	Percentage of area activity, received from foreign visitors
8	Area's earning diversity: effective number of business categories to represent 80th percentiles of the total number of transactions earned by area's businesses
9	Area's spending diversity: effective number of business categories to represent 80th percentiles of the total number of transactions made by area's residents
10	Area's business density
11	Percentage of gas/parking/toll spending of area's residents
12	Percentage of taxi spending of area's residents
13	Percentage of public transportation spending of area's residents
14	Percentage of cafe/restaurants spending of area's residents
15	Percentage of fast food spending of area's residents
16	Percentage of food spending of area's residents
17	Percentage of recreation spending of area's residents
18	Percentage of fashion/beauty/jewelry spending of area's residents
19	Percentage of medical spending of area's residents
20	Percentage of cultural spending of area's residents
21	Percentage of travel spending of area's residents
22	Percentage of area's residents nighttime spending
23	Percentage of area's residents weekend spending
24	Percentage of area's residents nighttime money spending
25	Percentage of area's residents weekend money spending
26	Percentage of area's nighttime earnings
27	Percentage of area's residents weekend earnings
28	Percentage of area's nighttime business transactions
29	Percentage of residents weekend transactions
30	Percentage of residents' activity performed outside the province
31	Percentage of out area residents' activity performed inside the province
32	Percentage of money spent by area residents outside the province
33	Area's residents spending in expensive locations.
