# Supplementary figures and images for: Socioeconomic characterization of regions through the lens of individual financial transactions
Source: PLoS One. 2017 Nov 30;12(11):e0187031. doi: 10.1371/journal.pone.0187031 (PMC5708635; doi:10.1371/journal.pone.0187031)

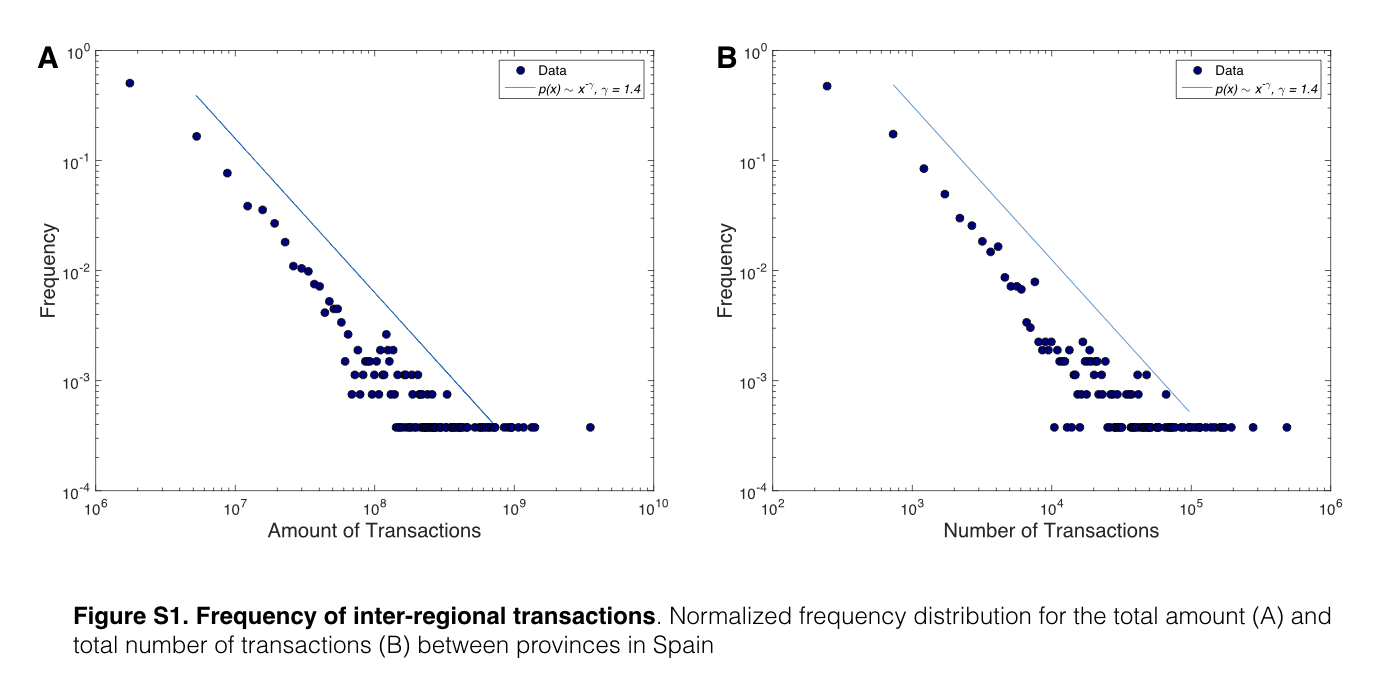

Supplement: S1 Fig — Normalized frequency distribution for the total amount (A) and total number of transactions (B) between provinces in Spain. (TIF) [file pone.0187031.s002.tif]

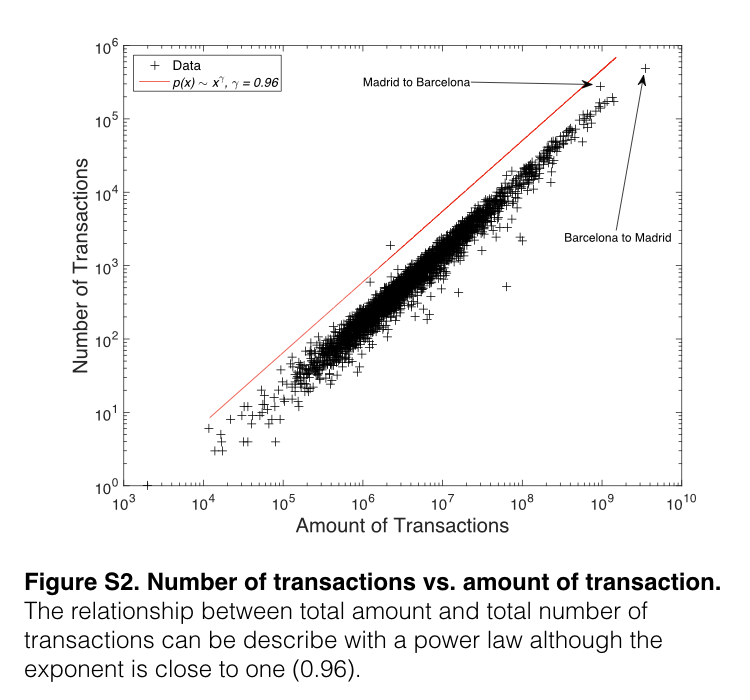

Supplement: S2 Fig — The relationship between total amount and total number of transactions can be describe with a power law although the exponent is close to one (0.96). (TIF) [file pone.0187031.s003.tif]

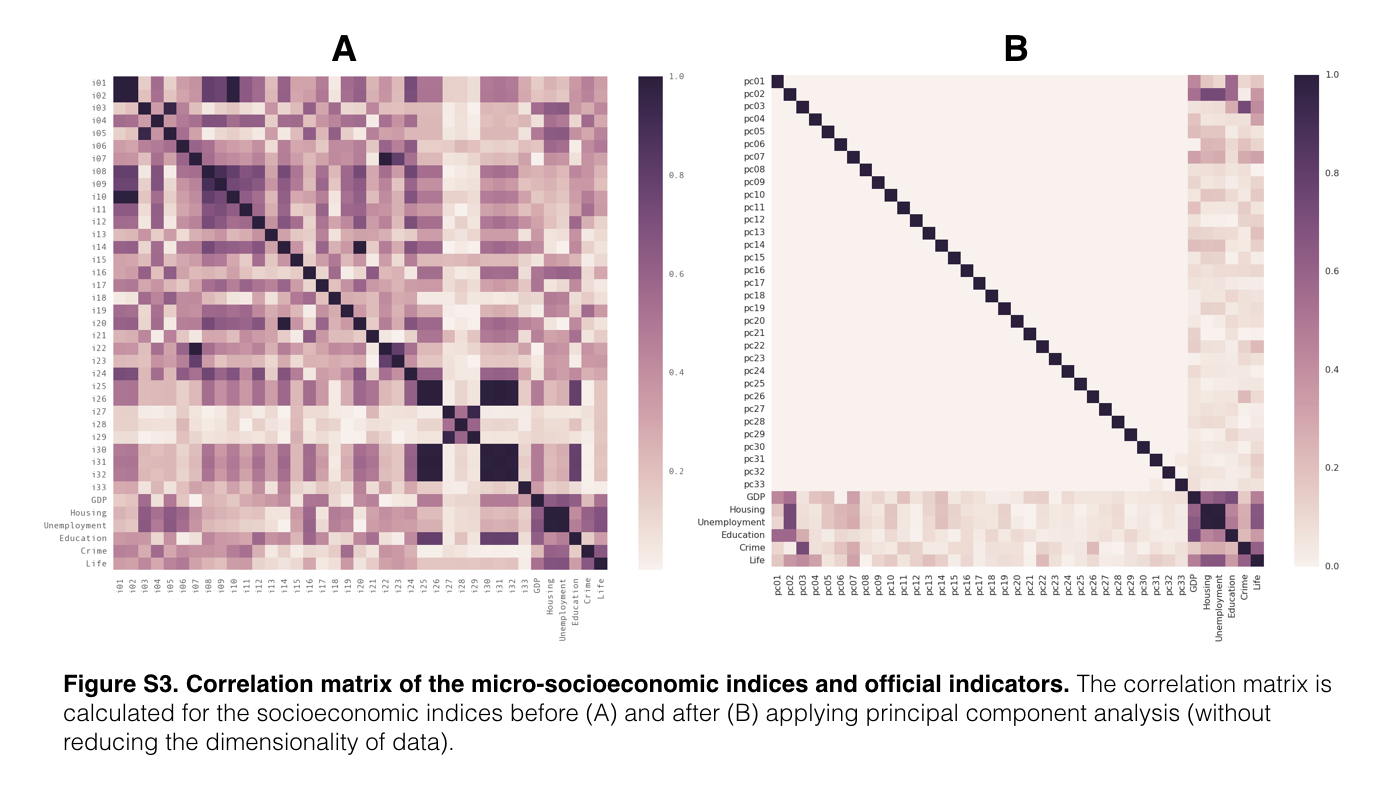

Supplement: S3 Fig — The correlation matrix is calculated for the socioeconomic indices before (A) and after (B) applying PCA (without reducing the dimensionality of data). (TIF) [file pone.0187031.s004.tif]

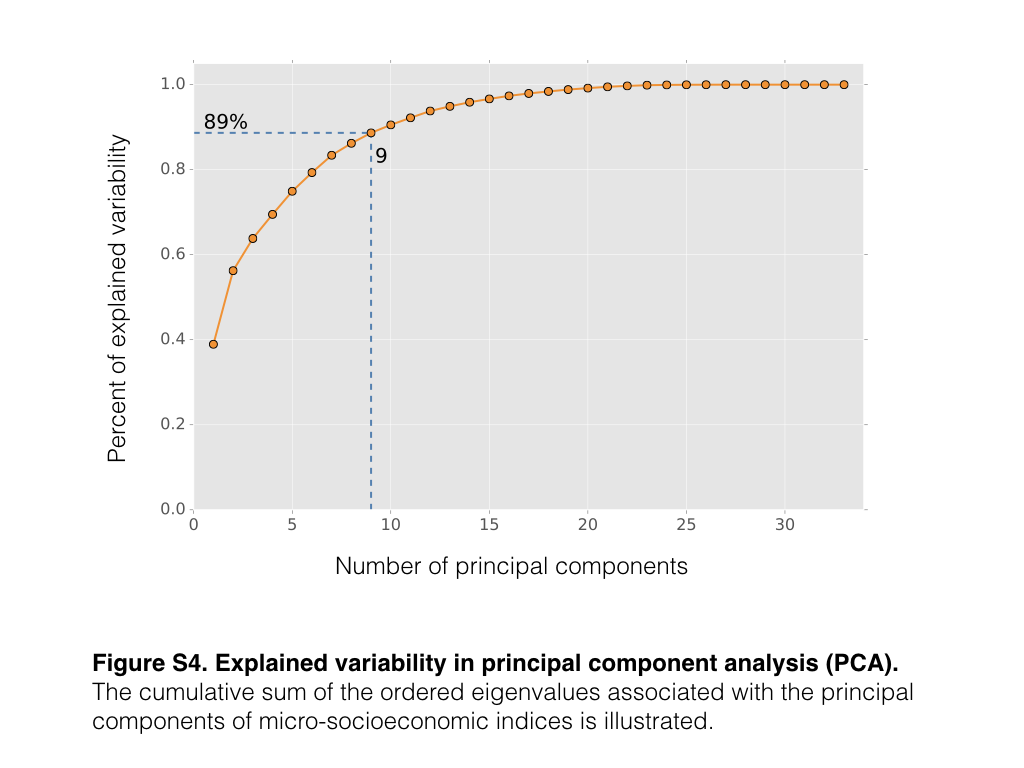

Supplement: S4 Fig — The cumulative sum of the ordered eigenvalues associated with the PCs of micro-socioeconomic indices is illustrated. (TIF) [file pone.0187031.s005.tif]

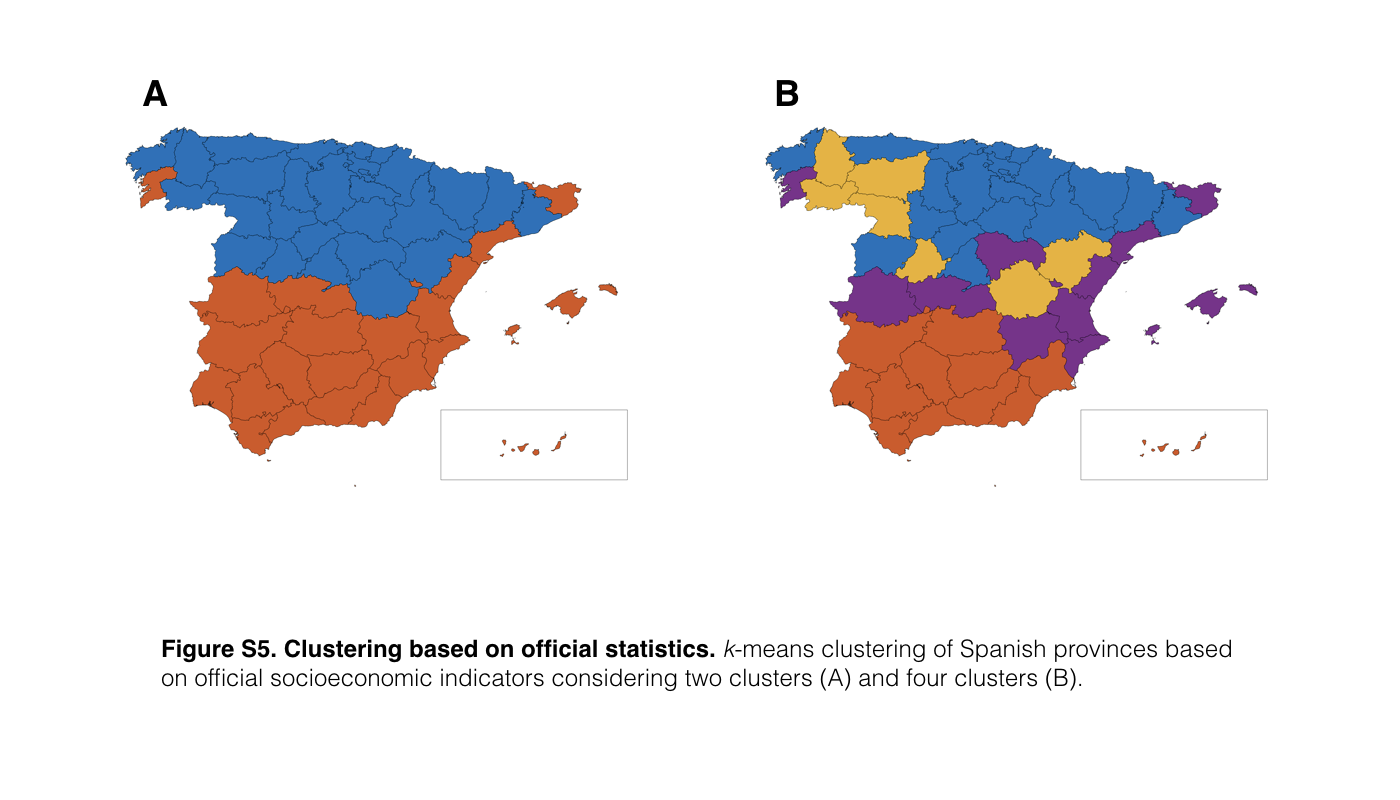

Supplement: S5 Fig — k-means clustering of Spanish provinces based on official socioeconomic indicators considering two clusters (A) and four clusters (B). (TIF) [file pone.0187031.s006.tif]

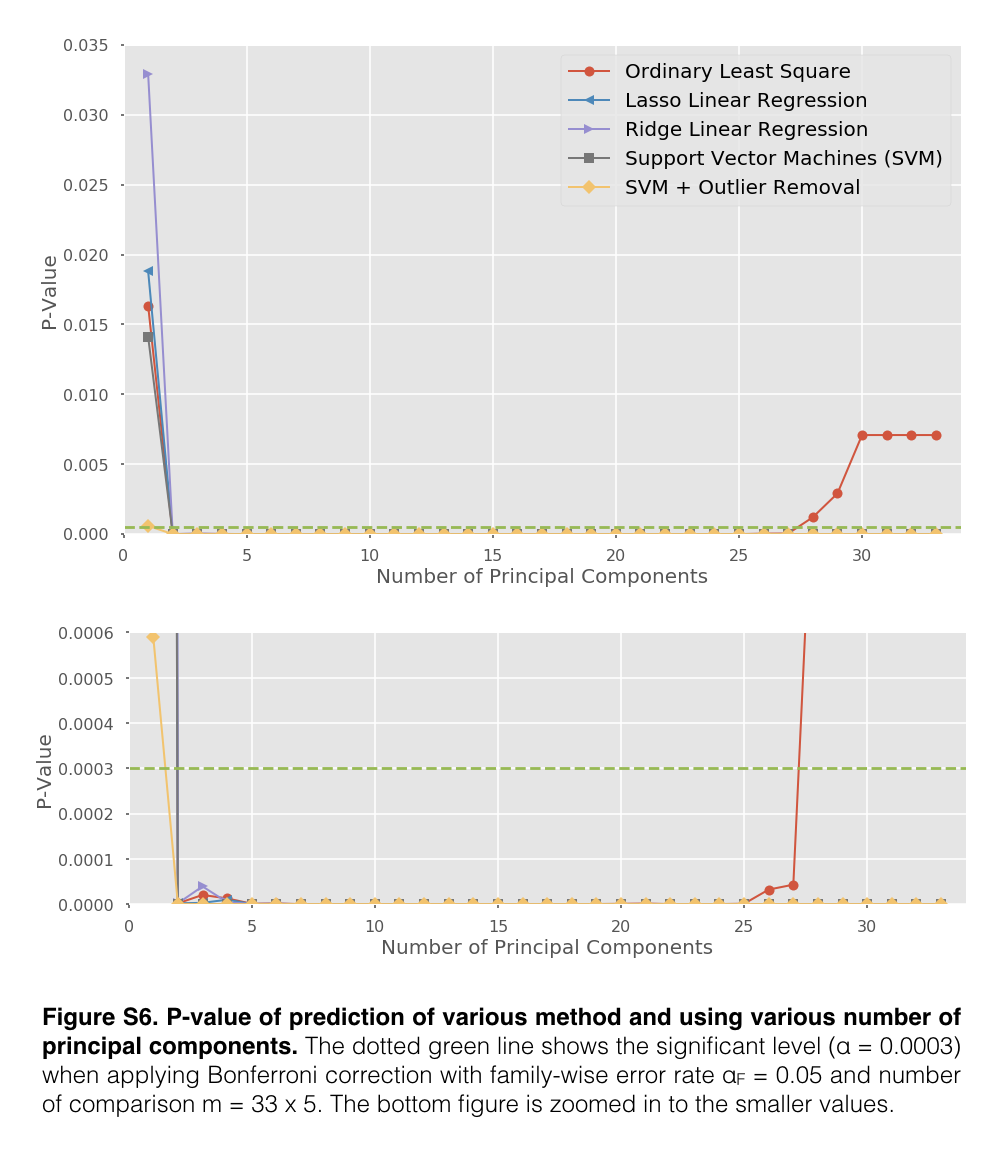

Supplement: S6 Fig — The dotted green line shows the significant level (α = 0.0003) when applying Bonferroni correction with family-wise error rate αF = 0.05 and number of comparison m = 33 × 5. The bottom figure is zoomed in to the smaller values. (TIF) [file pone.0187031.s007.tif]
